# Infant Hygiene

**Published:** 1859-01

**Authors:** 


Infant Hygiene.

A Society which has been organised under the title of the "Ladies' National Association for the Diffusion of Sanitary Knowledge," is issuing tracts on matters relating to health, written in a simple style, and published at a cheap rate, so as to be easily procured and understood by all classes. Among them we meet with four relating specially to the management of infants; bearing the titles, respectively, of —*How to Manage a Baby; How to Feed a Baby with the Bottle; The Evils of Wet Nursing—a Warning to Mothers;* and, *The Evils of Perambulators—a Word to Mothers.* The precepts laid down in these are in close accordance with the dictates of nature; and, if they are followed, it may be confidently expected that much both of the disease and of the mortality among children, which now is a disgrace to our civilisation, will be prevented. From the tract, *How to Manage a Baby,* we take as a specimen the remarks on Walking.

"People talk about 'teaching babies to walk;' but babies do not need teaching, for they will be sure to get up and walk, when their legs are strong enough, and it does them harm to do so before; in this, as in very many other things, babies would be all the better for being left to themselves. But this does not suit some mothers who are in a hurry to see their children walk; such mothers cannot rest content without putting their children into leading strings, or go-carts, or leading them with the hand. All that they generally get for their pains is the sight of their children's bandy legs and crooked ankles, caused by being forced to walk before their time. Who would be a baby?

"But, though baby should not be hurried in walking, it should be allowed to keep moving all day long, while it is awake, for the limbs cannot get strong unless they are used. The best plan is, to put a piece of soft matting and a piece of carpet on the floor, and put baby down upon them to stretch, roll, and tumble about like all other young creatures. If it has a ball or a rag doll to crawl about after, it will be 'as happy as the days are long,' and will, besides, be very little trouble, and be making its limbs strong, ready to walk by-and-bye. It is a great pity to accustom a baby to be nursed, for it only does it harm, and gives the mother a world of trouble into the bargain. . . . In the summer, it is a good plan to spread the matting and carpet on the grass in the garden, and put baby down on them, to use its limbs in the pure air and light. In short, wherever it is, and whatever it does, it should keep moving all the time. The birds, the beasts, the fish, and the creeping things are scarcely ever still five minutes together in the day time. Moving brings life and health to all things, babies among the rest."

The tract from which we have made this extract, contains also, advice arranged under the heads of Washing, Dress, Food, Cutting the Teeth, Sleep, Going Out-doors, Talking, Vaccination and Physic. Under the latter head, mothers are sensibly advised "to learn to keep themselves and their children well, and leave the curing illness to those who understand it."

